# A unified framework for deriving and visualizing soliton solutions in the paraxial nonlinear Schrödinger equation

**DOI:** 10.1038/s41598-025-07884-9

**Published:** 2025-09-26

**Authors:** Ali Al Khabyah, Ali Ahmad, Ali N. A. Koam, Adel Almarashi, Hakeem A Othman

**Affiliations:** 1 Department of Mathematics, College of Science, King Khalid University, 61413 Abha, Saudi Arabia; 2 Department of Computer Science, College of Engineering and Computer Science, Jazan University, Jazan, Saudi Arabia; 3 Department of Mathematics,College of Science, Jazan University, P.O. Box: 114, 45142 Jazan, Kingdom of Saudi Arabia; 4 Department of Mathematics, Albaydha University, Albaydha, Yemen

**Keywords:** Graphical visualization, Solitons, Paraxial nonlinear Schrödinger equation, Mathematics and computing, Physics

## Abstract

This research investigates the paraxial nonlinear Schrödinger equation commonly used in quantum mechanics, plasma physics, and nonlinear fiber optics. Employing the extended modified auxiliary equation mapping method, we obtained different soliton solutions, which were tested via Hamiltonian method of stability analysis. The dynamic behavior of the solutions was realized by making use of Stream Density graphs, 3D slice contour graphs, Linear graphs, Density linear graphs, and 2D graphs. The results obtained were tabulated systematically to ensure accuracy; therefore, this research would be of practical use in soliton dynamics and nonlinear wave propagation and can be useful in furtherance of mathematics and bio-mathematics as well as industrial research. The given model and soliton solutions can be efficaciously used to model pulse propagation in optical fibers, investigate energy localization in plasmas, and examine wave packet dynamics in quantum systems. Such uses highlight the practical relevance of paraxial nonlinear Schrödinger equation to promote technologies in telecommunications, fusion science, and nanoscale materials science.

## Introduction

Nonlinear evolution equations (NLEEs) find widespread application in many areas of applied sciences and engineering because of their capability to describe numerous intricate physical processes. Closed-form analytical developments of NLEEs are as much of mathematical interest as they are of value in understanding nonlinear wave behaviors of those processes in real-world systems. Such behaviors span many disciplines such as fiber optics, sound wave propagation, oceanography, shallow water hydrodynamics, fluid mechanics, nonlinear optics, plasma dynamics, neural networks, chaos, diffusion and reaction processes, solid-state dynamics, quantum dynamics, biological mathematics, and electromagnetic fields. One notable case is propagation of ultra-short electromagnetic pulses in nonlinear media, an n-dimensional process controlled by the subtle interaction of material dispersion, diffraction, and nonlinear behavior. This dynamic interaction may result in solitons (or light bullets), i.e., pulses of light that preserve their profile because of an exact balancing of dispersive and nonlinear effects. Solitons find useful applications in areas ranging from optical microscopy to data storage, laser acceleration of particles, Bose-Einstein condensation, and high-resolution signal transmission.

Another basic idea in nonlinear wave theory is modulational instability (MI), arising due to interaction between linear dispersion or diffraction and nonlinear self-action of waves. MI is a widespread mechanism governing wave development in many nonlinear systems^1–4^.

With increasing curiosity about nonlinear dynamics in numerous areas of science, research into NLEEs has emerged as an overarching theme. The majority of nonlinear behaviors in physical systems can be represented by these types of equations. Consequently, there has arisen an imperative to develop efficient means to discover exact solutions of NLEEs, particularly traveling wave solutions (TWS) since they model such behaviors in many physical systems.

Exact nonlinear Schr$\ddot{o}$dinger equation (NLSE) solutions have great significance in mathematical physics because of their capability of representing stable localized solitons of waves, known as optical solitons. Solitons result by carefully balancing dispersion and nonlinearity, and as such, the NLSE became an elemental model of wave propagation in nonlinear dispersive media. Besides its standard form, NLSE acts as a master framework generating many extended and varied models to address specific physical effects or limitations in diverse systems^5–9^. The generalized forms of NLSE include the Gerdjikov-Ivanov equation to account for self-steepening and Raman effects, Manakov model to describe propagation in birefringent fibers, Gabitov-Turitsyn equation to simulate dispersion-managed systems, Kundu-Eckhaus and Chen-Lee-Liu equations to include higher-order nonlinearities, and complex Ginzburg-Landau equation and Biswas-Milovic equation to model dissipative regimes. Other well-known models that result from or relate to NLSE include the Maxwell-Bloch system, Schr$\ddot{o}$dinger-Hirota equation to describe dispersive solitons, and integrable Sasa-Satsuma equation. Here, to gain insights about soliton propagation in different types of nonlinear wave guides, in this investigation, we consider an individual NLSE-based model and analyze its exact soliton solutions in specific physical regimes.

The paraxial nonlinear Schr$\ddot{o}$dinger equation has been the target of many investigations by using integration strategies, analytical methods, and numerical schemes. Some other new methods which can be applied recently to the paraxial nonlinear schr$\ddot{o}$dinger equation are the semi-inverse variational principle^10^, Lie symmetry analysis^11^, conservation law methods^12^, techniques for integrable nonlocal LPD equations^13,14^, improved Adomian decomposition method^15^, tanh expansion method^16^, modified simple equation method^17^, modified extended direct algebraic method^18^, undetermined coefficients method^19^, Riccati equation approach^20^. Nonlinear partial differential equations (NLPDEs) are fundamental across numerous scientific fields.

To accurately describe the physical processes represented by these equations, robust methodologies are required to obtain precise solutions. Selecting an appropriate approach for applying and interpreting PDEs is crucial in this research. Given that each PDE has unique characteristics, various effective approaches have been developed; however, no universal method suits all types of PDEs. Well-known techniques include the tanh expansion^21^, modified extended tanh expansion^22^, Adomian’s decomposition^23^, B$\ddot{a}$cklund transformation^24^, Painlev$\acute{e}$ expansion^25^, fractional homotopy analysis method^26^, Kudryashov’s method^27,28^, and the exponential rational function method^29^. Also noteworthy are the Khater approach^30^ and the enhanced generalized Riccati equation mapping method^31^. This study revisits the generalized auxiliary equation mapping technique, originally developed by Sirendaoreji^32^. Employing an appropriate auxiliary equation simplifies computations and facilitates the discovery of various types of exact solutions.

Recent scientific developments in nonlinear science have revealed the power of different analytical approaches to find exact analytical solutions of nonlinear evolution equations (NLEEs) in numerous physical models. Some examples include applications of exact analytical schemes to solitons in optics and nonlinear waves, fluid flow, fractional-order models, and geophysical models of waves, illustrating the accuracy and flexibility of such approaches^33–39^. Such studies greatly advance understanding of waves and represent powerful mathematics to describe intricate nonlinear processes.

The Extended Modified Auxiliary Equation Mapping (EMAMEM) method has some main advantages when compared to standard algorithms for computing soliton solutions. The method is capable of extracting a broader class of exact solutions, such as hybrid and complex solitons, by virtue of its flexible mapping mechanism. The technique is computational and reduces nonlinear PDEs to simpler lower-dimensional ODEs systematically, making it effective and applicable to both integrable and non-integrable systems. Furthermore, there is consistency between EMAMEM and tools for carrying out stability analysis, such that physically sound and mathematically exact solutions can be obtained. Its flexibility allows it to be an effective tool in the analysis of nonlinear wave dynamics in different scientific disciplines.

The paraxial nonlinear Schr$\ddot{o}$dinger equation (PNLSE) is considered in this research since it describes light-wave propagation in nonlinear media with paraxial approximation. The equation describes both linear diffraction and nonlinear processes like self-focusing and defocusing depending on medium properties. The more details and variations of this model can be found in^40–45^ The EMAMEM approach is used as an efficient tool to find soliton solutions and study how they behave when subject to stochastic perturbations. Stability of resulting solitary wave solutions is investigated with the Hamiltonian method, and findings are presented in tabular form to provide clarity and consistency. Consider the equation

1.1 $$\begin{aligned} i Q_y +\frac{\alpha }{2}Q_{xx}+\frac{\beta }{2} Q_{tt} -\gamma |Q|^2 Q=0. \end{aligned}$$

This is the general paraxial nonlinear Schr$\ddot{o}$dinger equation that takes into consideration both temporal dispersion and spatial variation, as well as nonlinear interaction between fields. It controls evolution of the complex envelope *q*(*x*, *y*, *t*) as it travels along the direction of propagation, *y*, considering spatial and temporal dispersive effects. The real parameters $\alpha$, $\beta$, and $\gamma$ in Eq. (1.1) refer to diffraction, dispersion, and Kerr nonlinearity, respectively. Eq. (1.1) takes the form of the elliptic NLSE when $\alpha \beta > 0$, and becomes the hyperbolic NLSE when $\alpha \beta < 0$^46,47^. This equation can remarkably model (2+1)-dimensional spatial dynamics in cubic Kerr media when group-velocity dispersion is neglected; when this occurs, *x* and *t* refer to transversal spatial coordinates, and *y* refers to longitudal propagation coordinate, and we assume $\alpha = \beta > 0$. The organization of the article is as follows:

Section “Introduction” provides a brief overview of the paraxial nonlinear schr$\ddot{o}$dinger equation. The algorithm used in the proposed method is explained in detail in Section “Methodology of extended modified AEM method”. Section “Stability analysis” demonstrates the stability of paraxial nonlinear schr$\ddot{o}$dinger equation. Graphical visualization of the solutions are presented in Section “Graph-based data visualization”. Section “Discussions and results” offer results and discussion along with concluding remark.

## Methodology of extended modified AEM method

We here apply newly formulated Extended Modified Auxiliary Equation Mapping Method (EMAMEM) by Seadawy^48^ to the paraxial nonlinear Schrödinger equation. The advanced analytical algorithm is employed to find a larger group of general and new traveling wave solutions. With the flexibility of EMAMEM, we can construct systematically exact solutions that uncover complex nonlinear wave dynamics of the system resulting from interaction of diffraction, dispersion, and Kerr nonlinearity.

The nonlinear partial differential equation is given as

2.1 $$\begin{aligned} M(w, w_t, w_x, w_y, w_{xx}, \ldots ) = 0, \end{aligned}$$

where $M$ is a polynomial in $w(x, y, t)$. To obtain the exact traveling wave solution, apply the traveling wave transformation in the following form:

2.2 $$\begin{aligned} w(x, y, t) = w(\xi ), \quad \xi = \sum _{i=0}^m \kappa _i x_i, \end{aligned}$$

where $\kappa _i$, $i = 0, 1, 2, \ldots , m$ are constants. Applying this transformation, Eq. (2.1) is simplified to a nonlinear ODE of the given form.

2.3 $$\begin{aligned} N(w, w', w'', w''', \ldots ) = 0, \end{aligned}$$

We have a nonlinear ordinary differential equation (NLODE), where $N$ denotes a polynomial in $w(\xi )$ along with its associated total derivatives $w',~w'',~w''',\ldots$. Next, we assume that $w(\xi )$ satisfies a general solution expressed as a series in terms of $\psi (\xi )$. Accordingly, we propose that the solution of the equation can be written in the following form:

2.4 $$\begin{aligned} w=w(\xi )=\sum ^{n}_{j=0}a_{j}\psi ^{j}(\xi )+\sum ^{-n}_{j=-1}b_{-j}\psi ^{j}(\xi )+\sum ^{n}_{j=2}c_{j}\psi ^{j-2}(\xi )\psi ^{'}(\xi )+\sum ^{n}_{j=1}d_{j}\bigg (\frac{\psi ^{'}(\xi )}{\psi (\xi )}\bigg )^{j}, \end{aligned}$$

where $a_k$, $b_k$, $c_k$, and $d_k$ are arbitrary constants that will be determined later. In this framework, $\psi (\xi )$ is assumed to satisfy the following generalized auxiliary equation:

2.5 $$\begin{aligned} \psi ^{'^{2}}=(\frac{d\psi }{d\xi })^{2}=\beta _{1}\psi ^{2}(\xi )+\beta _{2}\psi ^{3}(\xi )+\beta _{3}\psi ^{4}(\xi ), \end{aligned}$$

where $\beta _1$, $\beta _2$, $\beta _3$ are constants to be determined.

The transformation is given by:

2.6 $$\begin{aligned} z(x,y,t)= Q(\eta ) e^{i \phi }, \end{aligned}$$

where

2.7 $$\begin{aligned} \eta = x+y-\nu t, ~~~ \phi =\kappa x+\theta y -\nu t. \end{aligned}$$

Put Eq. (2.6) and Eq. (2.7) in Eq. (1.1), we get this,

2.8 $$\begin{aligned} A Q''(\eta )+B Q(\eta )-2 \gamma \kappa Q(\eta )^3, \end{aligned}$$

where $~~A= \alpha (\kappa -1) \nu ^2-1$ and $~~B= \kappa \left( \alpha (\kappa -1) \nu ^2-2 \theta +\kappa \right)$. Using the homogeneous balance principle, the non-linear term $Q^3$ with higher order derivative term *Q* then give $M+2=3M,$ which implies $~~M=1$. Therefore, we assume the solution takes the following form:

2.9 $$\begin{aligned} Q = a_1 \psi (\eta )+a_0+\frac{b_1}{\psi (\eta )}+\frac{d_1 \psi '(\eta )}{\psi (\eta )}, \end{aligned}$$

where $a_0$, $a_1$, $b_1$, and $d_1$ are constants to be determined.

**Family 1:**

2.10 $$\begin{aligned} a_{0}= & 0,~~a_{1}= -\frac{\sqrt{h} \sqrt{\alpha \kappa \nu ^2-\alpha \nu ^2-1}}{2 \sqrt{\gamma } \sqrt{\kappa }},~~b_{1}= 0,\nonumber \\ d_{1}= & -\frac{i \sqrt{-\alpha \kappa \nu ^2+\alpha \nu ^2+1}}{2 \sqrt{\gamma } \sqrt{\kappa }}.\nonumber \\ z_{1}(x,y,t)= & \Bigg ( \frac{1}{4} e^{(-i \phi )} \bigg ( \frac{ \frac{4 \sqrt{h} \kappa \left( j \tanh \left( E \left( \eta + \eta _0\right) \right) + 1\right) ^2 \left( \alpha (\kappa - 1) \nu ^2 - 2 \theta + \kappa \right) }{ g \sqrt{\alpha (\kappa - 1) \nu ^2 - 1}} - i E j \text {sech}\left( E \left( \eta + \eta _0\right) \right) ^2 \sqrt{2 - 2 \alpha (\kappa - 1) \nu ^2} }{\sqrt{\gamma } \sqrt{\kappa } \left( j \tanh \left( E \left( \eta + \eta _0\right) \right) + 1\right) }\bigg ) \Bigg ), \nonumber \\ \text {where} \quad \eta= & x+y-\nu t,\nonumber \\ E= & \sqrt{\frac{\kappa \left( \alpha (\kappa -1) \nu ^2-2 \theta +\kappa \right) }{\alpha (\kappa -1) \nu ^2-1}}.\nonumber \\ z_{2}(x,y, t)= & \Bigg ( e^{(-i \phi )} \bigg (-\frac{\sqrt{h} \sqrt{\frac{f}{h}} \sqrt{\alpha \kappa \nu ^2-\alpha \nu ^2-1} \left( \frac{F j}{G+\rho }+1\right) }{4 \sqrt{\gamma } \sqrt{\kappa }}-\frac{i \sqrt{-\alpha \kappa \nu ^2+\alpha \nu ^2+1} \left( \frac{\sqrt{f} G j}{G+\rho }-\frac{\sqrt{f} F^2 j}{(G+\rho )^2}\right) }{2 \sqrt{\gamma } \sqrt{\kappa } \left( \frac{F j}{G+\rho }+1\right) }\bigg ) \Bigg ), \nonumber \\ \text {where} \quad \eta= & x+y-\nu t, \nonumber \\ F= & \sinh \left( \sqrt{f} \left( \eta +\eta _0\right) \right) \nonumber \\ G= & \cosh \left( \sqrt{f} \left( \eta +\eta _0\right) \right) .\nonumber \\ z_{3}(x,y, t)= & \Bigg ( e^{(-i \phi )} \bigg ( \frac{f \sqrt{h} H \sqrt{\alpha \kappa \nu ^2-\alpha \nu ^2-1}}{2 \sqrt{\gamma } g \sqrt{\kappa }}+\frac{i \sqrt{f} j \sqrt{-\alpha \kappa \nu ^2+\alpha \nu ^2+1} \left( -F \sigma +G \rho \sqrt{\sigma ^2+1}+1\right) }{2 \sqrt{\gamma } H \sqrt{\kappa } (F+\sigma )^2}\bigg ) \Bigg ),\nonumber \\ \text {where} \quad \eta= & x+y-\nu t,\nonumber \\ F= & \sinh \left( \sqrt{f} \left( \eta +\eta _0\right) \right) ,\nonumber \\ G= & \cosh \left( \sqrt{f} \left( \eta +\eta _0\right) \right) ,\nonumber \\ H= & \frac{j \left( \cosh \left( \sqrt{f} \left( \eta +\eta _0\right) \right) +\rho \sqrt{\sigma ^2+1}\right) }{\sinh \left( \sqrt{f} \left( \eta +\eta _0\right) \right) +\sigma }+1. \end{aligned}$$

**Family 2:**

2.11 $$\begin{aligned} a_{0}= & \frac{1}{2} \left( \frac{\sqrt{2} \kappa ^2}{\sqrt{-\gamma \kappa ^2 (2 \theta -\kappa )}}-\frac{2 \sqrt{2} \theta \kappa }{\sqrt{-\gamma \kappa ^2 (2 \theta -\kappa )}}\right) ,a_{1}= -\frac{g}{2 \sqrt{2} \sqrt{\gamma \kappa ^3-2 \gamma \theta \kappa ^2}},\nonumber \\ b_{1}= & 0,~~d_{1}= 0. \nonumber \\ z_{4}(x,y, t)= & \Bigg ( e^{(-i \phi )} \bigg ( \frac{1}{2} \left( \frac{\sqrt{2} \kappa ^2}{\sqrt{-\gamma \kappa ^2 (2 \theta -\kappa )}}-\frac{2 \sqrt{2} \theta \kappa }{\sqrt{-\gamma \kappa ^2 (2 \theta -\kappa )}}\right) +\frac{f (j K+1)}{2 \sqrt{2} \sqrt{\gamma \kappa ^3-2 \gamma \theta \kappa ^2}}\bigg ) \Bigg ),\nonumber \\ \text {where} \quad \eta= & x+y-\nu t, \nonumber \\ K= & \tanh \left( \frac{1}{2} \sqrt{f} \left( \eta +\eta _0\right) \right) . \nonumber \\ z_{5}(x,y, t)= & \Bigg ( e^{(-i \phi )} \bigg ( \frac{1}{2} \left( \frac{\sqrt{2} \kappa ^2}{\sqrt{-\gamma \kappa ^2 (2 \theta -\kappa )}}-\frac{2 \sqrt{2} \theta \kappa }{\sqrt{-\gamma \kappa ^2 (2 \theta -\kappa )}}\right) -\frac{g H \sqrt{\frac{f}{h}}}{4 \sqrt{2} \sqrt{\gamma \kappa ^3-2 \gamma \theta \kappa ^2}}\bigg ) \Bigg ), \nonumber \\ \text {where} \quad \eta= & x+y-\nu t, \nonumber \\ H= & \frac{j \left( \cosh \left( \sqrt{f} \left( \eta +\eta _0\right) \right) +\rho \sqrt{\sigma ^2+1}\right) }{\sinh \left( \sqrt{f} \left( \eta +\eta _0\right) \right) +\sigma }+1.\nonumber \\ z_{6}(x,y,t)= & \Bigg ( e^{(-i \phi )} \bigg ( \frac{1}{2} \left( \frac{\sqrt{2} \kappa ^2}{\sqrt{-\gamma \kappa ^2 (2 \theta -\kappa )}}-\frac{2 \sqrt{2} \theta \kappa }{\sqrt{-\gamma \kappa ^2 (2 \theta -\kappa )}}\right) +\frac{f L}{2 \sqrt{2} \sqrt{\gamma \kappa ^3-2 \gamma \theta \kappa ^2}}\bigg ) \Bigg ), \nonumber \\ \text {where} \quad \eta= & x+y-\nu t, \nonumber \\ L= & \frac{j \left( \cosh \left( \sqrt{f} \left( \eta +\eta _0\right) \right) +\rho \sqrt{\sigma ^2+1}\right) }{\sinh \left( \sqrt{f} \left( \eta +\eta _0\right) \right) +\sigma }+1. \end{aligned}$$

**Family 3:**

2.12 $$\begin{aligned} a_{0}= & 0,~~a_{1}=\frac{i \sqrt{h}}{2 \sqrt{\gamma } \sqrt{\kappa }},~~b_{1}= 0,~~d_{1}= \frac{i}{2 \sqrt{\gamma } \sqrt{\kappa }}. \nonumber \\ z_{7}(x,y, t)= & \Bigg ( e^{(-i \phi )} \bigg ( \frac{i \sqrt{f} j M^2}{4 \sqrt{\gamma } \sqrt{\kappa } N}-\frac{i f \sqrt{h} N}{2 \sqrt{\gamma } g \sqrt{\kappa }}\bigg ) \Bigg ), \nonumber \\ \text {where} \quad \eta= & x+y-\nu t, \nonumber \\ M= & \text {sech} \left( \frac{1}{2} \sqrt{f} \left( \eta +\eta _0\right) \right) ,\nonumber \\ N= & j \tanh \left( \frac{1}{2} \sqrt{f} \left( \eta +\eta _0\right) \right) +1.\nonumber \\ z_{8}(x,y, t)= & \Bigg ( e^{(-i \phi )} \bigg ( \frac{i \left( \frac{\sqrt{f} G j}{G+\rho }-\frac{\sqrt{f} F^2 j}{(G+\rho )^2}\right) }{2 \sqrt{\gamma } \sqrt{\kappa } \left( \frac{F j}{G+\rho }+1\right) }+\frac{i \sqrt{h} \sqrt{\frac{f}{h}} \left( \frac{F j}{G+\rho }+1\right) }{4 \sqrt{\gamma } \sqrt{\kappa }}\bigg ) \Bigg ), \nonumber \\ \text {where} \quad \eta= & x+y-\nu t,\nonumber \\ F= & \sinh \left( \sqrt{f} \left( \eta +\eta _0\right) \right) ,\nonumber \\ G= & \cosh \left( \sqrt{f} \left( \eta +\eta _0\right) \right) . \nonumber \\ z_{9}(x,y, t)= & \Bigg ( e^{(-i \phi )} \bigg ( -\frac{i f \sqrt{h} \left( \frac{j \left( G+\rho \sqrt{\sigma ^2+1}\right) }{F+\sigma }+1\right) }{2 \sqrt{\gamma } g \sqrt{\kappa }}-\frac{i \sqrt{f} j \left( -F \sigma +G \rho \sqrt{\sigma ^2+1}+1\right) }{2 \sqrt{\gamma } \sqrt{\kappa } (F+\sigma )^2 \left( \frac{j \left( G+\rho \sqrt{\sigma ^2+1}\right) }{F+\sigma }+1\right) }\bigg ) \Bigg ), \nonumber \\ \text {where} \quad \eta= & x+y-\nu t,\nonumber \\ F= & \sinh \left( \sqrt{f} \left( \eta +\eta _0\right) \right) ,\nonumber \\ G= & \cosh \left( \sqrt{f} \left( \eta +\eta _0\right) \right) . \end{aligned}$$

**Family 4:**

2.13 $$\begin{aligned} a_{0}= & 0,~~a_{1}=0,~~b_{1}= 0,~~d_{1}= \frac{\sqrt{-2 \theta +\kappa +1}}{\sqrt{2 \gamma f-\gamma \kappa }},~~ \alpha = \frac{2 f-2 \theta \kappa +\kappa ^2}{(\kappa -1) \nu ^2 (2 f-\kappa )}. \nonumber \\ z_{10}(x,y, t)= & \Bigg ( e^{-i \phi } \bigg ( \frac{\sqrt{f} j \sqrt{-2 \theta + \kappa + 1} \, \text {sech} \left( \frac{1}{2} \sqrt{f} \left( \eta + \eta _0\right) \right) ^2}{2 \sqrt{2 \gamma f - \gamma \kappa } \left( j \tanh \left( \frac{1}{2} \sqrt{f} \left( \eta + \eta _0\right) \right) + 1\right) }\bigg ) \Bigg ), \nonumber \\ \text {where} \quad \eta= & x + y - \nu t. \nonumber \\ z_{11}(x,y, t)= & \Bigg ( e^{-i \phi } \bigg ( \frac{\sqrt{-2 \theta +\kappa +1} \left( \frac{\sqrt{f} G j}{G+\rho }-\frac{\sqrt{f} F^2 j}{(G+\rho )^2}\right) }{\sqrt{2 \gamma f-\gamma \kappa } \left( \frac{F j}{G+\rho }+1\right) }\bigg ) \Bigg ), \nonumber \\ \text {where} \quad \eta= & x + y - \nu t, \nonumber \\ F= & \sinh \left( \sqrt{f} \left( \eta +\eta _0\right) \right) , \nonumber \\ G= & \cosh \left( \sqrt{f} \left( \eta +\eta _0\right) \right) . \nonumber \\ z_{12}(x,y, t)= & \Bigg ( e^{-i \phi } \bigg ( -\frac{\sqrt{f} j \sqrt{-2 \theta +\kappa +1} \left( -F \sigma +G \rho \sqrt{\sigma ^2+1}+1\right) }{(F+\sigma )^2 \sqrt{2 \gamma f-\gamma \kappa } \left( \frac{j \left( G+\rho \sqrt{\sigma ^2+1}\right) }{F+\sigma }+1\right) }\bigg ) \Bigg ), \nonumber \\ \text {where} \quad \eta= & x + y - \nu t, \nonumber \\ F= & \sinh \left( \sqrt{f} \left( \eta +\eta _0\right) \right) , \nonumber \\ G= & \cosh \left( \sqrt{f} \left( \eta +\eta _0\right) \right) . \end{aligned}$$

## Stability analysis

We investigated the stability of traveling wave solutions to Eq. (2.8) by using the Hamiltonian method where Hamiltonian-system (HS) momentum is given by

3.1 $$\begin{aligned} U=\int ^{\infty }_{-\infty }\frac{z(x)^{2}}{2}dx, \end{aligned}$$

with $z(x,y,t)$ denoting the traveling wave solution. The condition of stability mandates that

3.2 $$\begin{aligned} \frac{\partial ~U}{\partial ~\nu }>0, \end{aligned}$$

where $\nu$ represents the velocity of waves. The Eqs. (3.1) and (3.2) are used to establish specific regimes and ranges of parameters where the traveling waves of the HS behave in a stable manner. By using these requirements of stability in their corresponding valid ranges, we identified the stability behavior of resulting solutions as given in Table 1.

**Table 1:** Stability Analysis of $z_{i}(x,y,t)~~~i=1,2,3,\dots ,12.$.

No	Solution	Stability	Values of the variables
1	$z_{1}(x,y,t)$	Stable	$\theta = 1.9,\alpha = 1.7,\nu = 1.6,\kappa = 1.5,j = 1,\eta _0 = 1.3,h = 1.1, g = 1.2$, *x*, $t~\varepsilon ~[-3,3]$
2	$z_{2}(x,y,t)$	Stable	$\eta _0 = 1.3,h = 1.1,g = 1.2,\gamma = 1.4,\rho = 1,f = 1.8, y = 2, k = 1$, *x*, $t~\varepsilon ~[-3,3]$
3	$z_{3}(x,y,t)$	Stable	$j = 1,\eta _0 = 1.3,h= 1.1, g = 1.2,\gamma = 1.4,\rho = 1,f = 1.8,\sigma = 1,y = 2,k = -1$, *x*, $t~\varepsilon ~[-3,3]$
4	$z_{4}(x,y,t)$	Stable	$\alpha = 1.7,\nu = 1.6,\kappa = 1.5,j = 1,\eta _0 = 1.3,h = 1.1, g = 1.2,\gamma = 1.4$, *x*, $t~\varepsilon ~[-3,3]$
5	$z_{5}(x,y,t)$	Stable	$\alpha = 1.7,\nu = 1.9,\kappa = 1.5,j = 1,\eta _0 = 1.5, h = 1.1,g = 1.2,\rho = 1,f = 1.8$, *x*, $t~\varepsilon ~[-3,3]$
6	$z_{6}(x,y,t)$	Unstable	Singular solution
7	$z_{7}(x,y,t)$	Stable	$\nu = 1.5,\kappa = 1.5,j = 1,\eta _0 = 1.9,h = 1.1, g = 1.2,\gamma = 1.4,f = 1.3$, *x*, $t~\varepsilon ~[-3,3]$
8	$z_{8}(x,y,t)$	Stable	$\eta _0 = 1.5,h = 1.1, g = 1.2,\gamma = 1.4,\rho = 1,f = 1.8, y = 2, k = -1$, *x*, $t~\varepsilon ~[-2,2]$
9	$z_{9}(x,y,t)$	Stable	$\theta = 1.9,\alpha = 1.7,\nu = 1.2,\kappa = 1.5,j = 1,\eta _0 = 1.9,h = 1.1, g = 1.2$, *x*, $t~\varepsilon ~[-2,2]$
10	$z_{10}(x,y,t)$	Stable	$\nu = 1.2,\kappa = 1.5,j = 1,\eta _0 = 1.1, h = 1.6, g = 1.2,\gamma = 1.4, f = 1.8$, *x*, $t~\varepsilon ~[-2,2]$
11	$z_{11}(x,y,t)$	Stable	$\eta _0 = 1.3,h = 1.1, g = 1.2,\gamma = 1.4,\rho = 1,f = 1.8, y = 2, k = -1$, *x*, $t~\varepsilon ~[-2,2]$
12	$z_{12}(x,y,t)$	Stable	$\theta = 1.9,\alpha = 1.7,\nu = 1.6,\kappa = 1.5,j = 1,\eta _0 = 1.3,h = 1.1, g = 1.2$, *x*, $t~\varepsilon ~[-3,3]$

## Graph-based data visualization

**Fig. 1:**
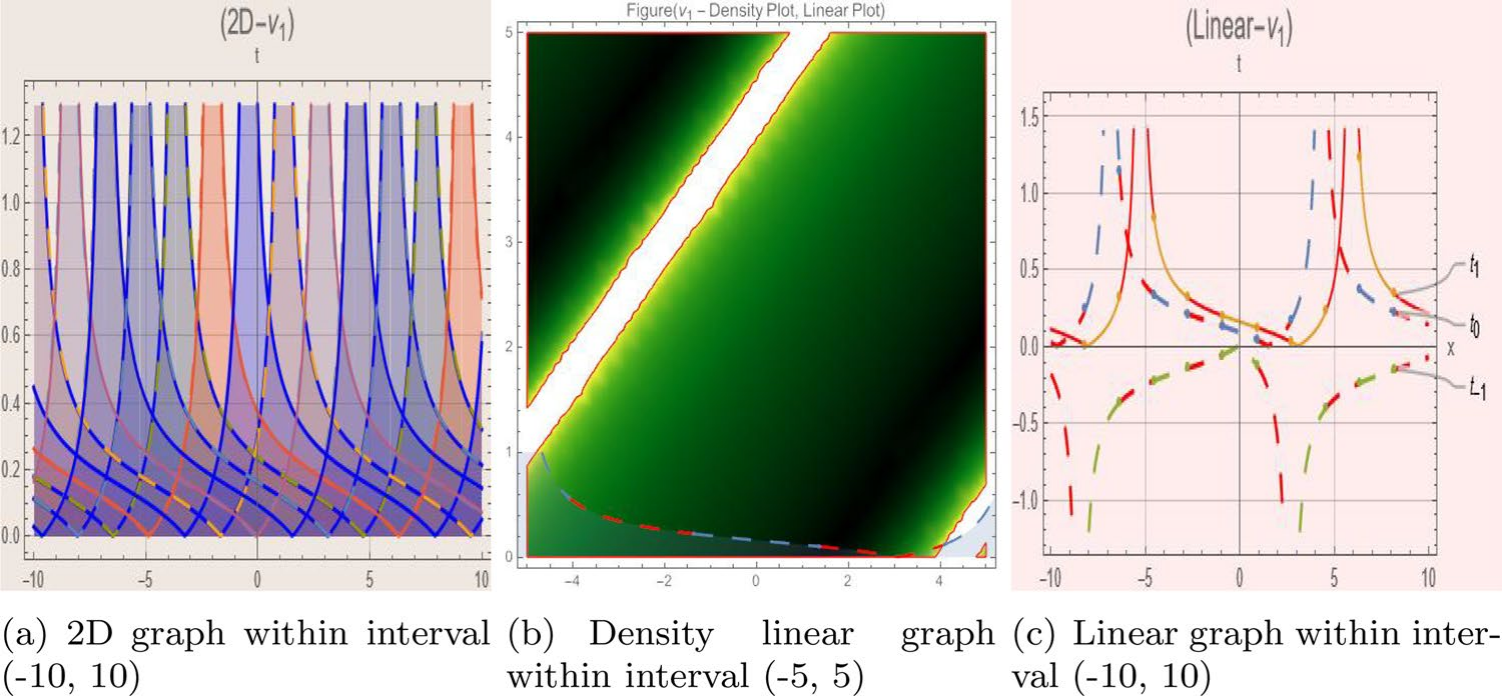
Graphic representation of $z_{1}(x,y,t)$ in various dimensions with $\theta = 1.9,\alpha = 1.7,\nu = 1.6,\kappa = 1.5,j = 1,\eta _0 = 1.3,h = 1.1, g = 1.2$, *x*, $t~\varepsilon ~[-3,3]$.

**Fig. 2:**
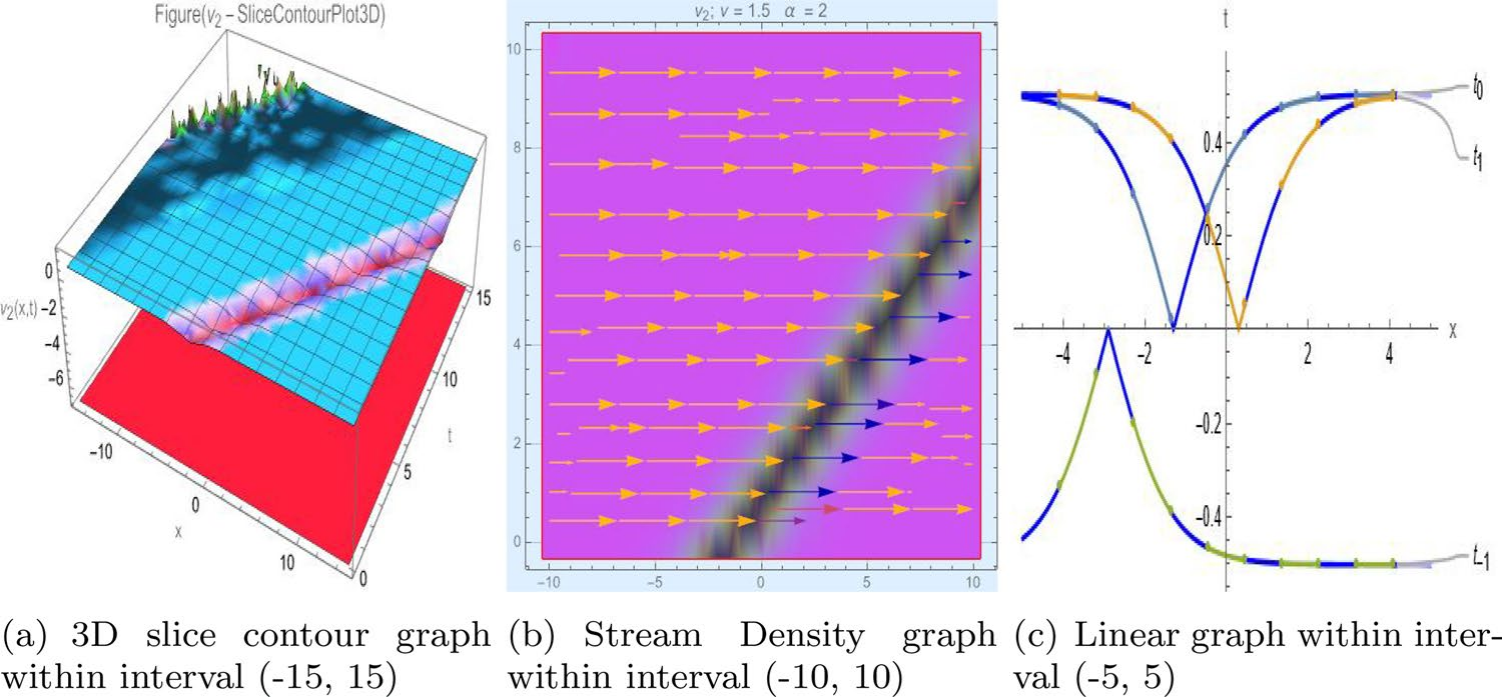
Graphic representation of $z_{2}(x,y,t)$ in various dimensions with $\eta _0 = 1.3,h = 1.1,g = 1.2,\gamma = 1.4,\rho = 1,f = 1.8, y = 2, k = 1$, *x*, $t~\varepsilon ~[-3,3]$.

**Fig. 3:**
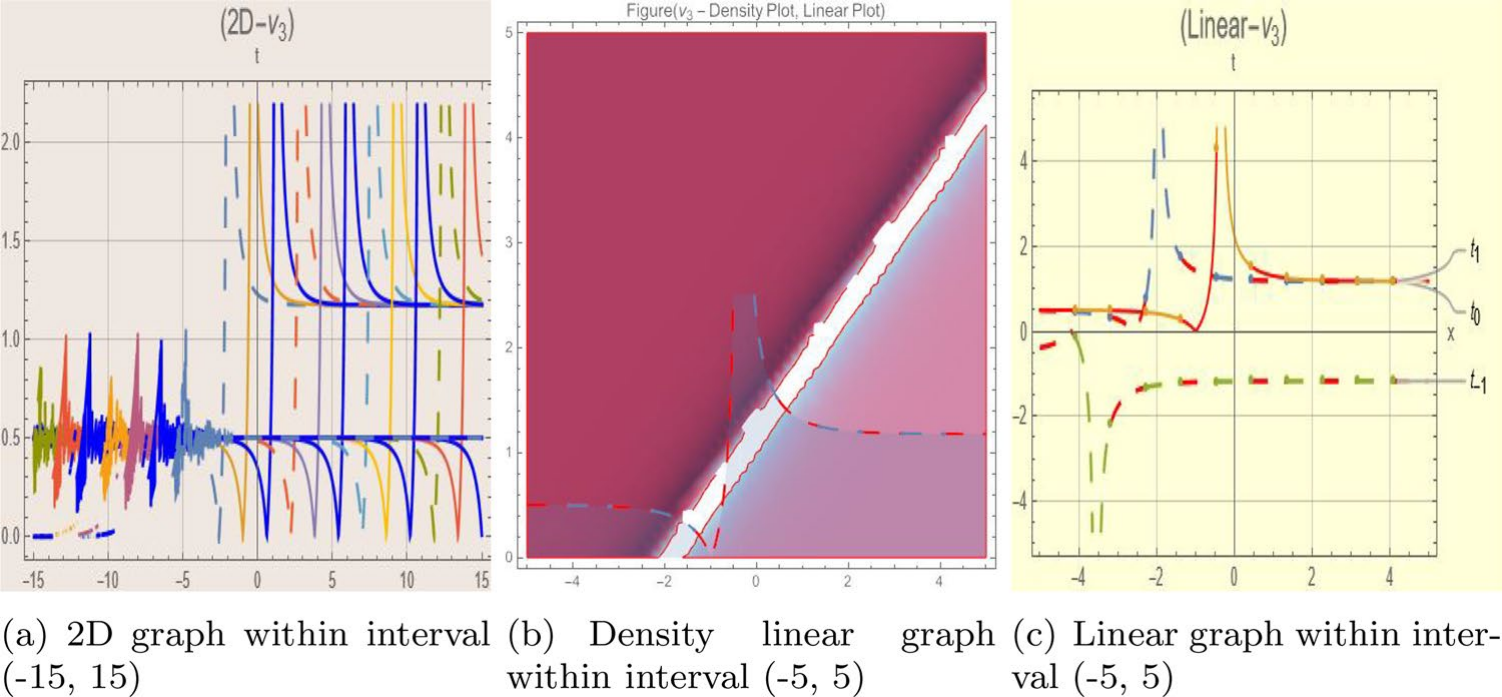
Graphic representation of $z_{3}(x,y,t)$ in various dimensions with$j = 1,\eta _0 = 1.3,h= 1.1, g = 1.2,\gamma = 1.4,\rho = 1,f = 1.8,\sigma = 1,y = 2,k = -1$, *x*, $t~\varepsilon ~[-3,3]$.

**Fig. 4:**
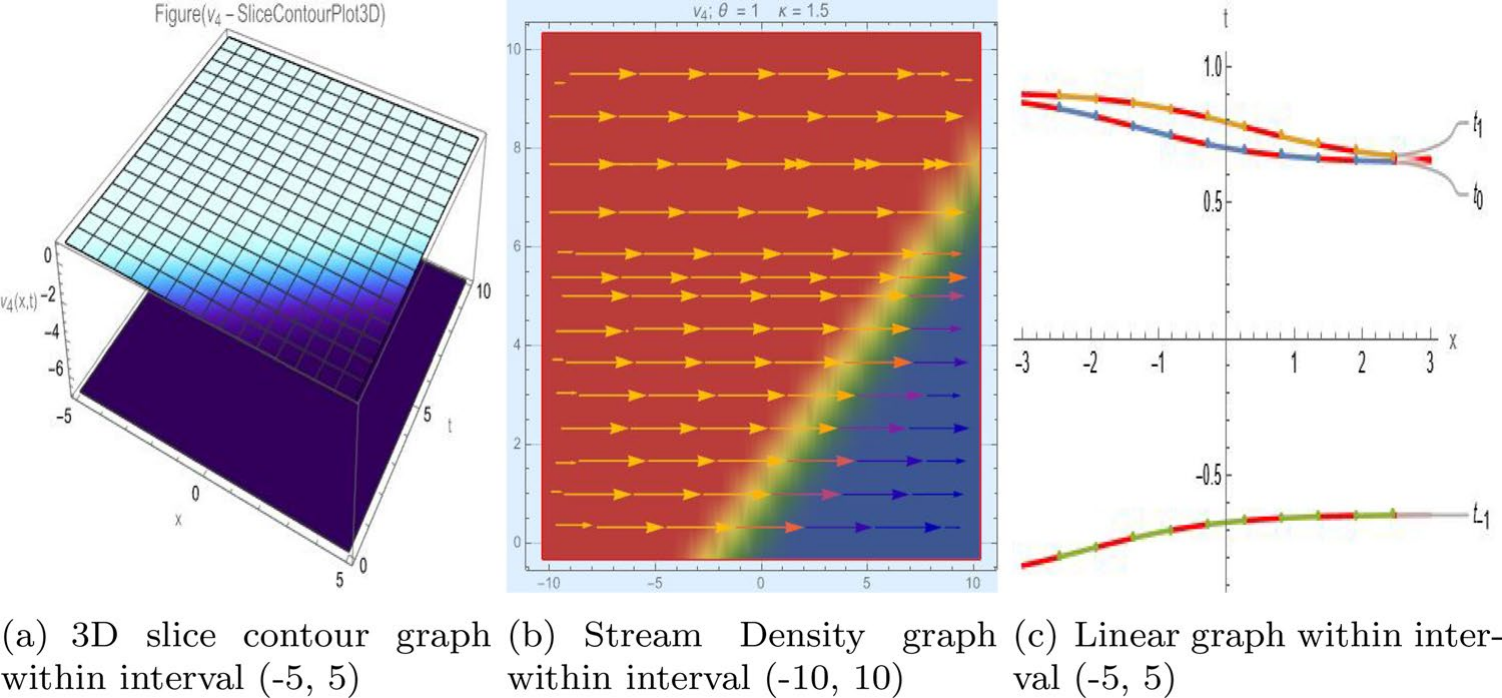
Graphic representation of $z_{4}(x,y,t)$ in various dimensions with $\alpha = 1.7,\nu = 1.6,\kappa = 1.5,j = 1,\eta _0 = 1.3,h = 1.1, g = 1.2,\gamma = 1.4$, *x*, $t~\varepsilon ~[-3,3]$.

**Fig. 5:**
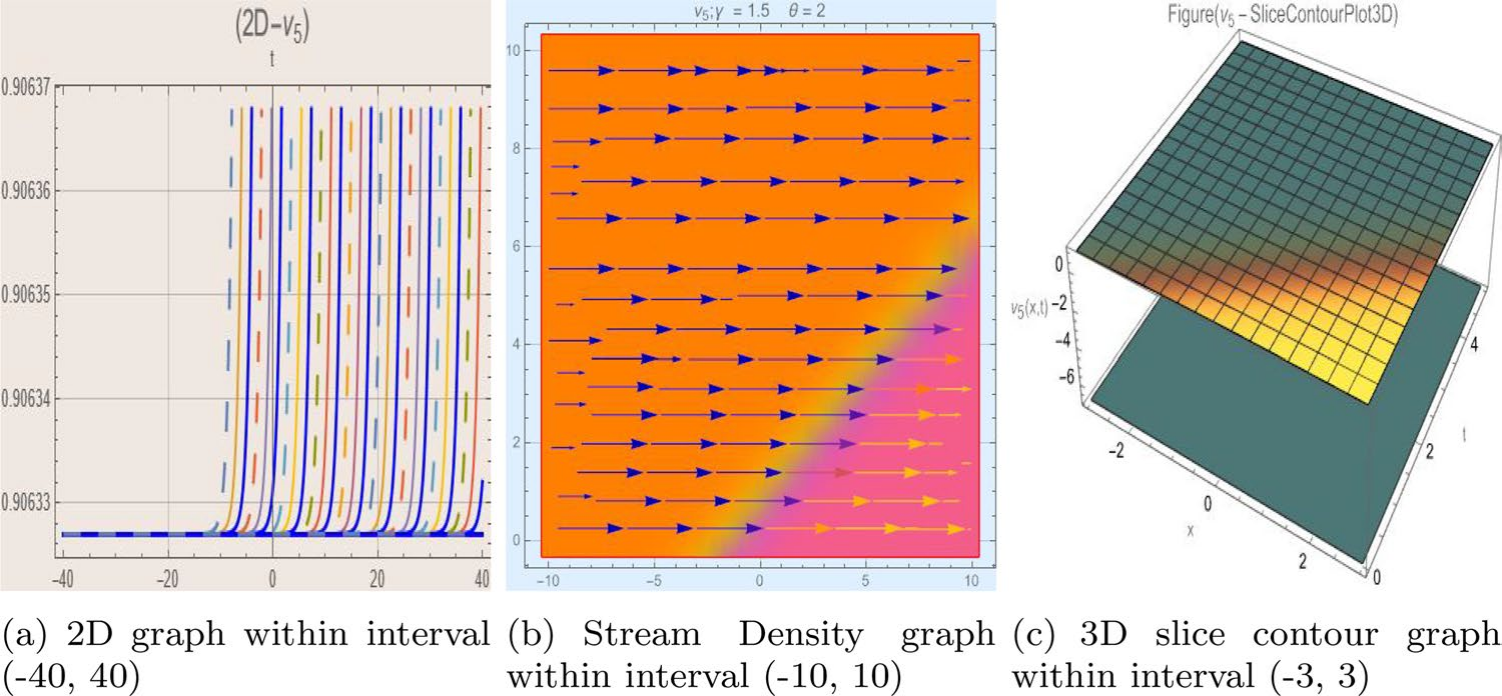
Graphic representation of $z_{5}(x,y,t)$ in various dimensions with $\alpha = 1.7,\nu = 1.9,\kappa = 1.5,j = 1,\eta _0 = 1.5, h = 1.1,g = 1.2,\rho = 1,f = 1.8$, *x*, $t~\varepsilon ~[-3,3]$.

**Fig. 6:**
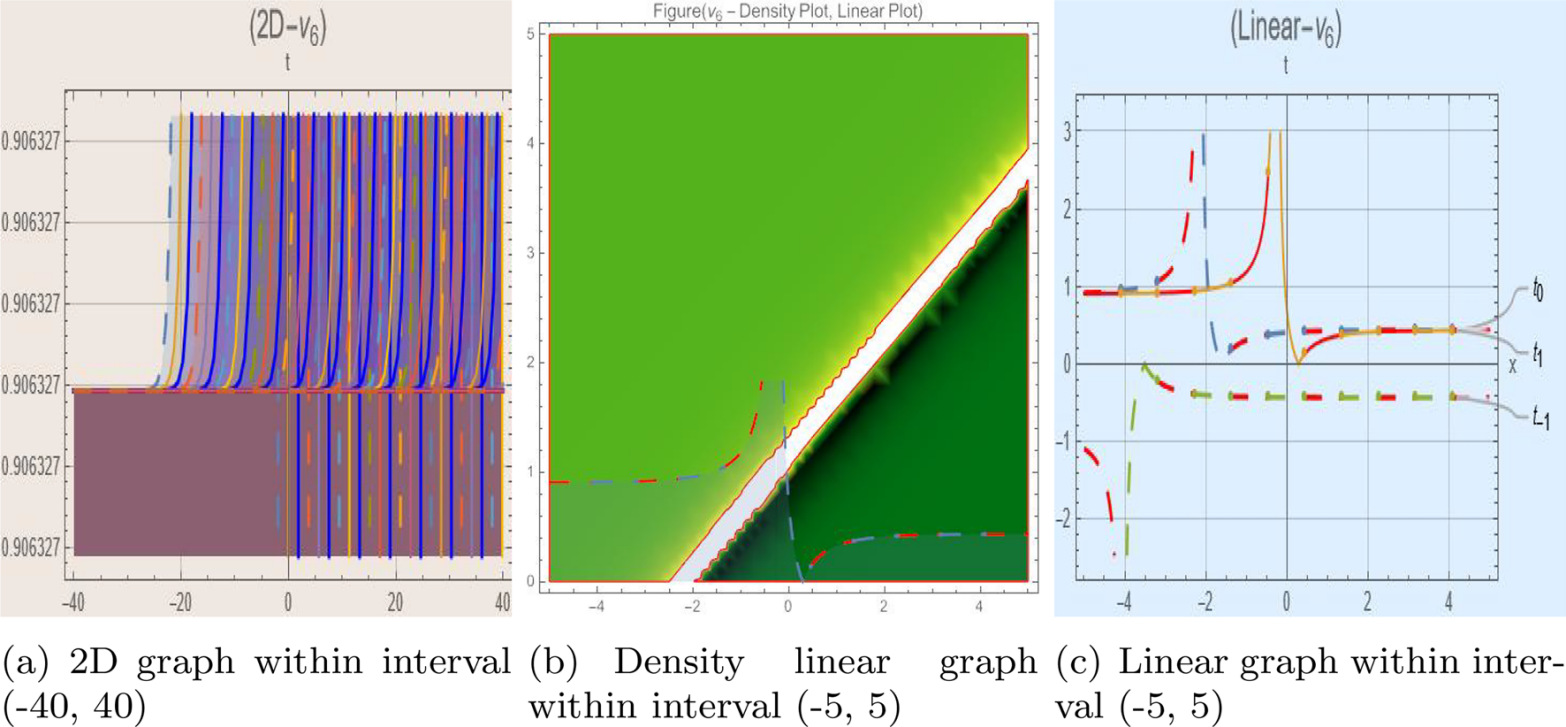
Graphic representation of $z_{6}(x,y,t)$ in various dimensions with $h = 1.1,g = 1.2,\gamma = 1.4,\rho = 1,f = 1.8,\sigma = 1,y = 2,k = 1$, *x*, $t~\varepsilon ~[-3,3]$.

**Fig. 7:**
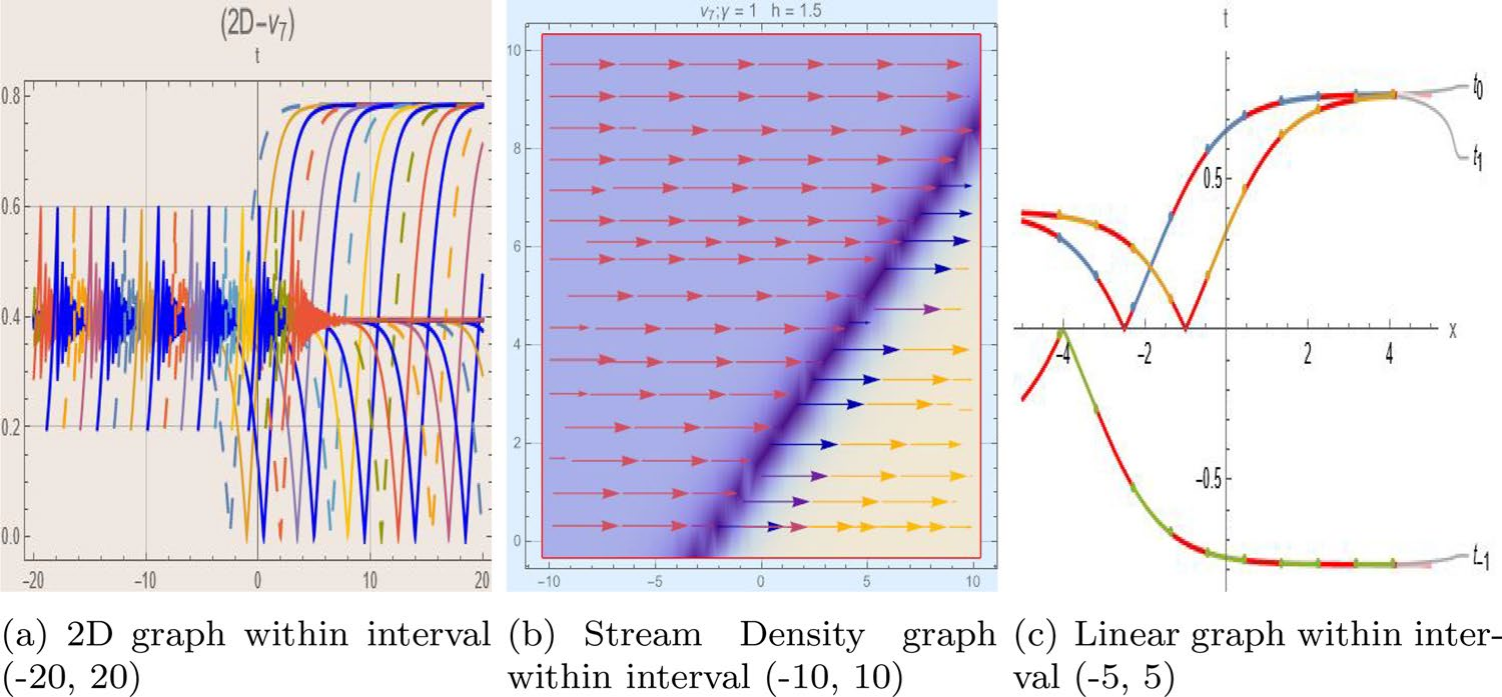
Graphic representation of $z_{7}(x,y,t)$ in various dimensions with $\nu = 1.5,\kappa = 1.5,j = 1,\eta _0 = 1.9,h = 1.1, g = 1.2,\gamma = 1.4,f = 1.3$, *x*, $t~\varepsilon ~[-3,3]$.

**Fig. 8:**
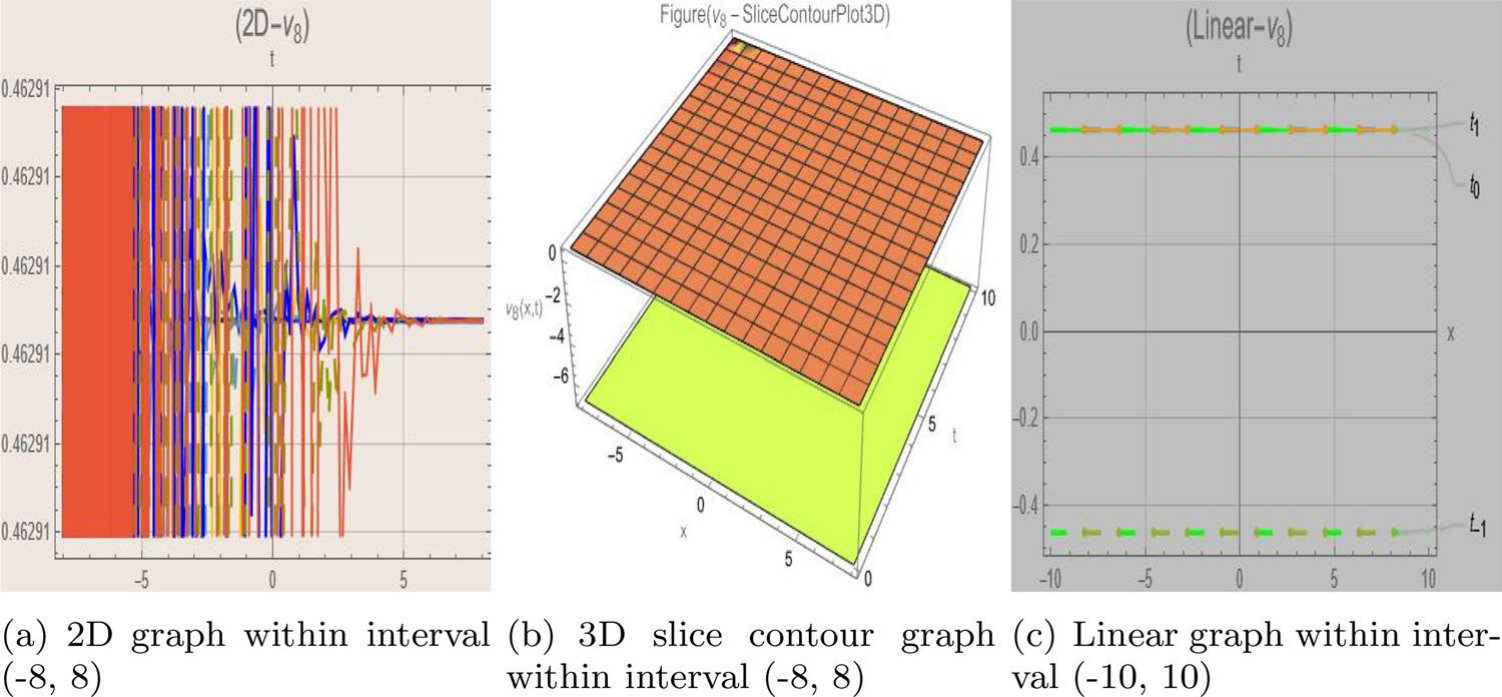
Graphic representation of $z_{8}(x,y,t)$ in various dimensions with $\eta _0 = 1.5,h = 1.1, g = 1.2,\gamma = 1.4,\rho = 1,f = 1.8, y = 2, k = -1$, *x*, $t~\varepsilon ~[-2,2]$.

**Fig. 9:**
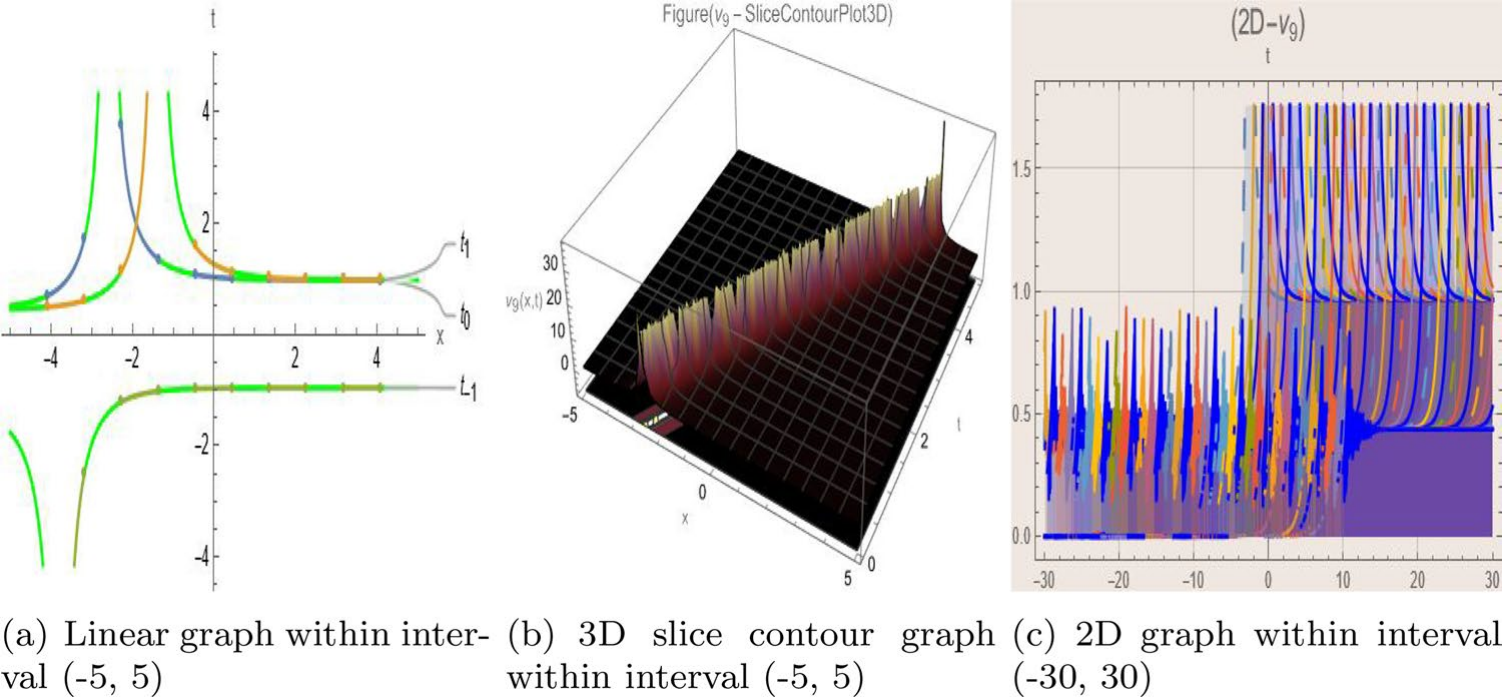
Graphic representation of $z_{9}(x,y,t)$ in various dimensions with $\theta = 1.9,\alpha = 1.7,\nu = 1.2,\kappa = 1.5,j = 1,\eta _0 = 1.9,h = 1.1, g = 1.2$, *x*, $t~\varepsilon ~[-2,2]$.

**Fig. 10:**
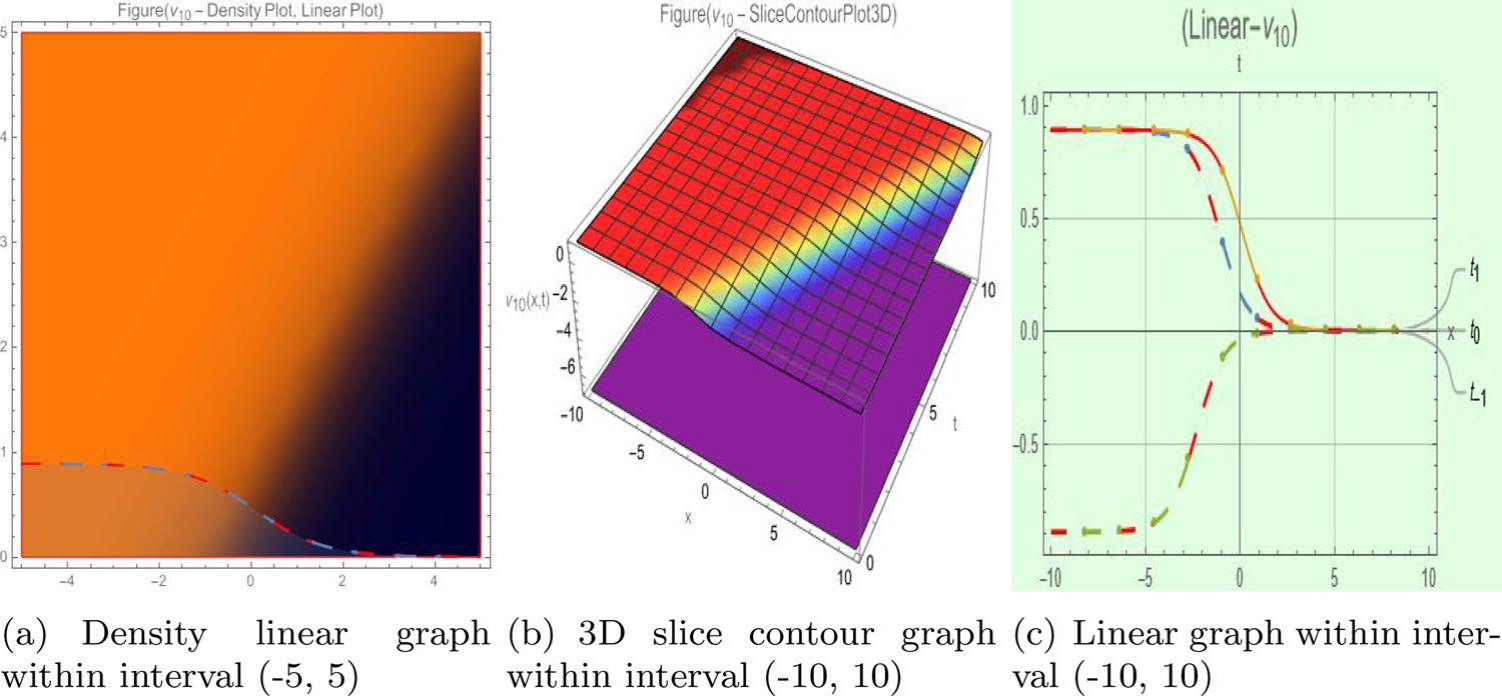
Graphic representation of $z_{10}(x,y,t)$ in various dimensions with $\nu = 1.2,\kappa = 1.5,j = 1,\eta _0 = 1.1, h = 1.6, g = 1.2,\gamma = 1.4, f = 1.8$, *x*, $t~\varepsilon ~[-2,2]$.

**Fig. 11:**
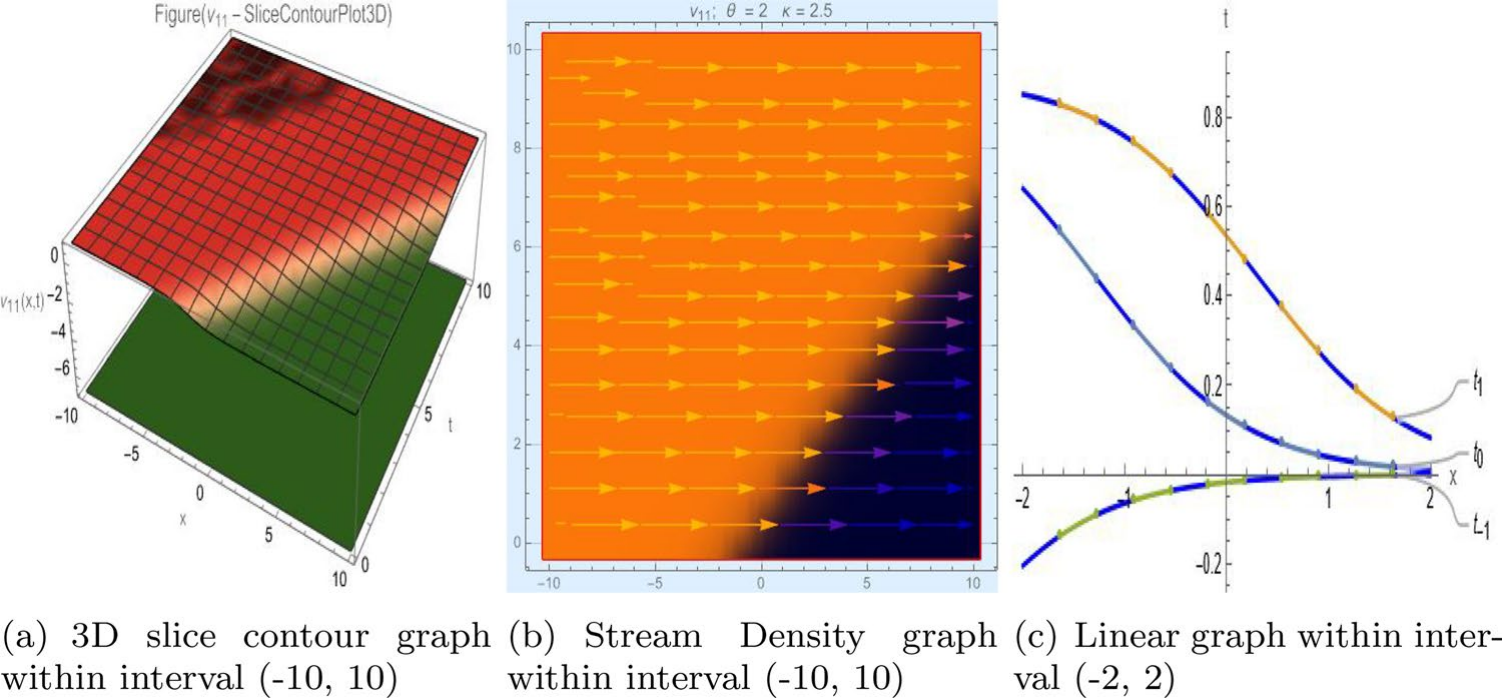
Graphic representation of $z_{11}(x,y,t)$ in various dimensions with $\eta _0 = 1.3,h = 1.1, g = 1.2,\gamma = 1.4,\rho = 1,f = 1.8, y = 2, k = -1$, *x*, $t~\varepsilon ~[-2,2]$.

**Fig. 12:**
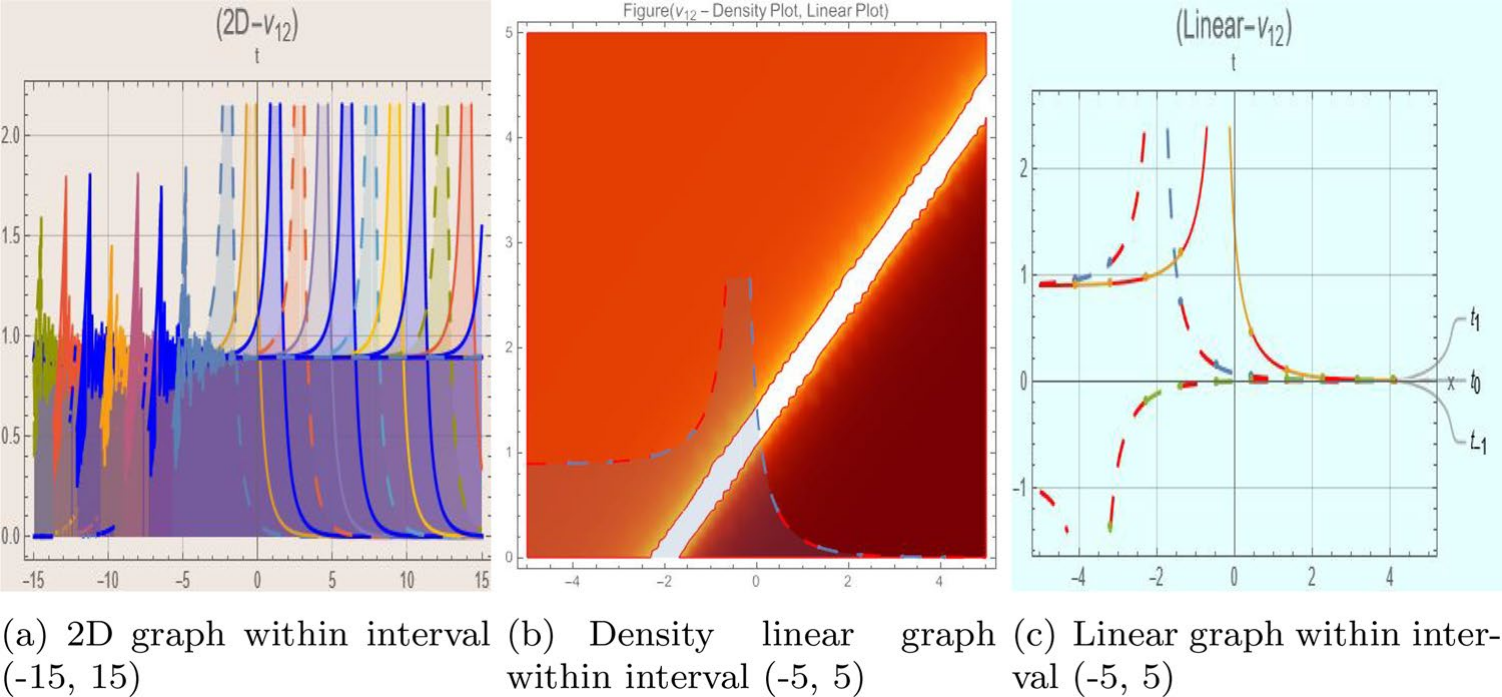
Graphic representation of $z_{12}(x,y,t)$ in various dimensions with $\theta = 1.9,\alpha = 1.7,\nu = 1.6,\kappa = 1.5,j = 1,\eta _0 = 1.3,h = 1.1, g = 1.2$, *x*, $t~\varepsilon ~[-3,3]$.

Using Mathematica 14.0, this part presents graphical representations of our recently discovered solutions in several dimensions (various kinds of rational, trigonometric, mixed, and hyperbolic functions). The objective is to have a deeper understanding of the physical interpretation of the model presented by Eq. (2.8). Figs. 1, 2, 3, 4, 5, 6, 7, 8, 9, 10, 11 and 12 give 2D graph, 3D slice contour, density linear, stream density and linear plots which describes the behaviour of the system and types of the family of soliton solutions presented by Eq. (2.10)–Eq. (2.13).

The Figs. 1, 2, 3, 4, 5, 6, 7, 8, 9, 10, 11 and 12 depict varieties of soliton profiles obtained as exact solutions of nonlinear evolution equations $z_i(x,y,t)$. From them, we can see bright, dark, and singular solitons, visualized as 2D line graphs, 3D surface plots, density plots, and stream flow plots. Each figure depicts unique spatiotemporal dynamics within different regimes of parameters, exhibiting localized maxima, intensity minima, and steep wavefronts-illustrating the soliton solution’s capability to capture intricate nonlinear dynamics.

Moreover, Figs. 1, 2, 3, 4, 5, 6, 7, 8, 9, 10, 11 and 12 can be clearly classified by analysis to reflect soliton types according to their graphical characteristics. Bright solitons, as depicted by localized peaks or humps against flat or zero backgrounds, can be seen in Figs. 1, 2, 3, 7, 9, 11 and 12, where energy concentration and maxima of wave amplitude stand out. On the other hand, dark solitons, indicated by localized minima of intensity or by a notch in an otherwise continuous wave background, can be seen in Figs. 4, 5, 6, 8, and 10 and can be determined by depressions in amplitude or in density, highlighting differences in soliton nature between different solutions. This analysis can be verified using the following condition on the families of solutions, bright solitons arise when $\gamma > 0$ and $\alpha (\kappa - 1)\nu ^2 < 1$, typically featuring a $\text {sech}^2(...)$ profile. In contrast, dark solitons occur for $\gamma < 0$ with $\alpha (\kappa - 1)\nu ^2 > 1$, characterized by a $\tanh (...)$ profile.

## Discussions and results

This section identifies and emphasizes the differences and commonalities between the recently obtained sets of solutions and those already reported in the literature by using traditional analytical approaches to the paraxial nonlinear Schr$\ddot{o}$dinger equation.

- Firstly, our method as presented in Eq. (2.4) uses a set of three parameters and results in a structurally different and new formulation. This structural difference is one of the most outstanding differences with previous efforts.
- Secondly, we use computational software like “Mathematica 14.0” to represent the multidimensional behaviors of the newly obtained solutions by specifying different sets of constant values $a_j$, $b_{-j}$, $c_j$, and $d_j$.
- Specifically, Eq. (2.4) can give rise to a rich variety of analytical solutions, such as rational, trigonometric, hyperbolic, and combination function types, embodying the flexibility and applicability of the presented method. Additionally, the results obtained by applying “extended trial equation method”, “modified auxiliary expansion method”, “modified extended mapping method”, “modified extended direct algebraic method”, “improved simple equation method”, and “modified extended auxiliary equation mapping scheme” differ sharply from results that we have recently obtained. The discrepancies can be observed by comparing with results presented in^40–45^.

## Concluding remarks

This study was dedicated to the paraxial nonlinear Schr$\ddot{o}$dinger equation, which describes the interaction of spatial diffraction, temporal dispersion, and Kerr nonlinearity in (2+1)-dimensional media. The parameters $\alpha$, $\beta$, and $\gamma$ signify these physical phenomena, respectively, and control soliton solution evolution along the propagation coordinate. With the aid of the Extended Modified Auxiliary Equation Mapping (EMAMEM) method, numerous exact bright and dark soliton solutions were obtained. The tradeoff between dispersion and nonlinearity in these solutions identifies dominating factors behind energy localization and stable pulse propagation in instances such as optical fibers, plasmas, and quantum media.

The Hamiltonian method was employed to confirm the stability of these solitons, and their dynamic evolution was realized with precise graphical analyses. In contrast to traditional approaches such as Inverse Scattering Transform or Hirota’s method, EMAMEM is more versatile and effective in generating varied and physically realistic solutions. The results do not just advance nonlinear wave equations mathematically, and they provide useful information for practical application in nonlinear quantum systems, plasma physics, and photonics as well.

The paraxial nonlinear Schr$\ddot{o}$dinger model is an effective tool to study the evolution of wave packets subject to the joint action of dispersion, diffraction, and nonlinearity. Aside from exact solitons, dynamic behavior of the model can be investigated using advanced tools like sensitivity analysis, chaotic analysis, and Lyapunov exponents, measuring the system’s sensitivity to initial values. Such tools unveil deeper understanding of the system’s predictability and resilience and possible transition to complicated, unstable regimes.

## Data Availability

The data that support the findings of this study is within the manuscript.
